# Thyroid function, glycemic control, and diabetic nephropathy in patients with type 2 diabetes over 24 months: prospective observational study

**DOI:** 10.1186/s12902-023-01393-4

**Published:** 2023-07-10

**Authors:** Hiroshi Iwakura, Tomoyuki Takagi, Hidefumi Inaba, Asako Doi, Yoko Ueda, Shinsuke Uraki, Ken Takeshima, Yasushi Furukawa, Tatsuya Ishibashi, Shuhei Morita, Shohei Matsuno, Masahiro Nishi, Hiroto Furuta, Taka-aki Matsuoka, Takashi Akamizu

**Affiliations:** 1 The First Department of Medicine, Wakayama Medical University, 811-1 Kimiidera, Wakayama, 641-8509 Japan; 2 Wakayama City Medical Association Seijinbyo Center, 2-1-2 Tebira, Wakayama, Wakayama, 640-8319 Japan

**Keywords:** Type 2 diabetes, Thyroid function, Glycemic control, Diabetic nephropathy

## Abstract

**Background:**

The higher prevalence of thyroid dysfunction in type 1 diabetes patients has been well established, whereas it is a matter of debate whether that is also observed in type 2 diabetes patients. This study was conducted to reveal whether higher prevalence of thyroid dysfunction is observed in patients with type 2 diabetes.

**Methods:**

We examined thyroid functions and thyroid autoantibodies in 200 patients with type 2 diabetes and 225 controls, with 24 months follow up for those with type 2 diabetes.

**Results:**

Serum free triiodothyronine (fT3) levels and fT3/free thyroxine (fT4) ratio were significantly lower, while fT4 levels were significantly higher in patients with type 2 diabetes. The number of patients with thyroid dysfunction or patients positive for thyroid autoantibodies were not different between the two groups. The fT3/fT4 ratio was positively and negatively correlated with serum c-peptide and HbA1c levels, respectively, suggesting that the difference can be attributable to insulin resistance and diabetic control. In the follow-up observation, we found no significant correlation between basal thyrotropin (TSH), fT3, fT4 or fT3/fT4 ratio with the amounts of changes of HbA1c levels at 12 or 24 months after the basal measurements. There was a negative relationship between TSH levels and eGFR at baseline measurements, but TSH levels did not seem to predict future decline of eGFR levels. No relationship was observed between urine albumin/ g‧cre levels and thyroid function.

**Conclusion:**

Thyroid dysfunction and thyroid autoantibodies were not different in prevalence between patients with type 2 diabetes and controls, although in patients with type 2 diabetes, the fT3/fT4 ratio was decreased. Basal thyroid function did not predict future diabetes control or renal function within 24 months of follow-up.

## Background

Thyroid dysfunction is one of the most common health problems. According to the Japan Thyroid Association, the prevalence of thyroid disease including thyroid nodules is estimated to be between 5 and 7 million (approximately 4 to 5.5% of the general population) in Japan. Diabetes is also a highly prevalent disease, with estimated prevalence in Japan is 18.7% in men and 9.3% in women according to the National Health and Nutrition Examination Survey 2018 [1]. Due to such high prevalence of these two disorders, a number of patients are likely to have both diabetes and thyroid dysfunction concurrently. The prevalence of thyroid dysfunction is suggested to be disproportionately higher in patients with diabetes than in the general population [2–16].

When the prevalence of thyroid dysfunction in patients with diabetes is examined, patients with type 1 and type 2 diabetes should be treated separately. Patients with type 1 diabetes are well-known to have an increased risk of developing other autoimmune disorders [17], including autoimmune thyroid disease [18]. The etiology of comorbidity between type 1 diabetes and autoimmune thyroid disease is at least partially attributable to the common genetic background susceptibility to these two diseases [17].

The prevalence of thyroid dysfunction has been widely reported to be high in patients with type 2 diabetes [2, 7, 8, 11, 13, 16, 19], but others argue that the incidence of thyroid dysfunction is not dissimilar to that of the general population [15, 20–23]. Thyroid hormones have a wide variety of physiological actions, some of which affect glucose metabolism. These actions include stimulation of general metabolism, absorption of glucose from the gut, and lipolysis. Administration of thyroid hormone to healthy volunteers induces insulin resistance and hyperglycemia [24], and patients with thyrotoxicosis show oxyhyperglycemia and insulin resistance [25]. Hyperthyroidism can induce diabetes, which in Japan is classified as diabetes by other causes, although it may sometimes be misclassified as type 2 diabetes when hyperthyroidism is overlooked. Conversely, hypothyroidism causes obesity due to the reduced general metabolic rate, which induces insulin resistance, eventually leading to glucose intolerance [26–28].

There are no known reports that directly compare the prevalence of thyroid dysfunction in patients with and without type 2 diabetes in Japan. Furthermore, although several reports have suggested the association between subclinical hypothyroidism and diabetic nephropathy [29–31] and retinopathy [32, 33], these studies are basically cross-sectional, however, and there are no prospective studies.

In this study, the prevalence of thyroid dysfunction in patients with type 2 diabetes are compared with that in control subjects without diabetes. We analyze the relationship between thyroid function and metabolic parameters and examine the effects of basal thyroid function on future glucose control and diabetic nephropathy. Additionally, the occurrences thyroid disease in diabetic patients are monitored during the course of observation.

## Methods

### Patients and study design

We recruited 203 patients with type 2 diabetic who visited the Wakayama Medical University Hospital between January 2018 and July 2019. Eligible were the patients aged 20 years or older and meeting the diagnostic criteria of diabetes developed by the Japan Diabetes Society [34] at least once, with or without medications including any oral hypoglycemic agents, GLP-1 analogues, and insulin. We excluded the patients with previously-known thyroid diseases including autoimmune thyroid diseases and thyroid nodules, those on medication which may affect thyroid function (e.g., iodine, amiodarone, or lithium), those who diagnosed with type 1 diabetes or diabetes due to other causes, and those that were pregnant. Patients with diabetes were evaluated at the time of enrollment, and then at 12 and 24 months thereafter. For non-diabetic controls, we enrolled 225 subjects who visited the Wakayama City Medical Association Center for a medical check-up. Control subjects had never met the diagnostic criteria of diabetes, were at least 20 years old, and had no known thyroid diseases. Unlike those with diabetes, controls were evaluated only at the time of enrollment due to the difficultly in follow up. The study protocol was approved by the Wakayama Medical University Ethical Committee and written informed consent was obtained from all patients. The study was performed in accordance with the Declaration of Helsinki.

### Laboratory measures

Serum thyrotropin (TSH), free triiodo-thyronine (fT3), free thyroxine (fT4), anti-thyroglobulin antibody (TgAb), anti-thyroid peroxidase antibody (TPOAb), anti-thyrotropin receptor antibody (TRAb), insulin, c-peptide (CPR), cardiac troponin I, and ferritin levels were determined by CL AIA-PACK® reagents (Tosoh corporation, Tokyo Japan). Serum osteocalcin levels were determined by ST AIA-PACK® osteocalcin (Tosho corporation, Tokyo, Japan). Serum total cholesterol (T-cho), LDL, HDL cholesterol, triglyceride (TG), plasma glucose, HbA1c, urine albumin, creatinine, and protein levels were measured by routine lab assays at the Wakayama Medical University Hospital.

### Statistical analysis

Statistical analysis was conducted by JMP Pro 14. Statistical significance of differences in mean values of baseline characteristics and thyroid function were assessed by Student’s *t*-test. Baseline medications rate, thyroid dysfunction rate, and thyroid autoantibody positive rate were assessed by chi-square test. Multiple regression analysis with backward stepwise selection was used to assess the importance of the valuables to determine the levels of fT3, fT4, fT3/fT4 ratio and HbA1c. All values were expressed as the mean ± S.E. Differences of *P* < 0.05 were considered significant.

## Results

### Baseline characteristics

**Fig. 1:**
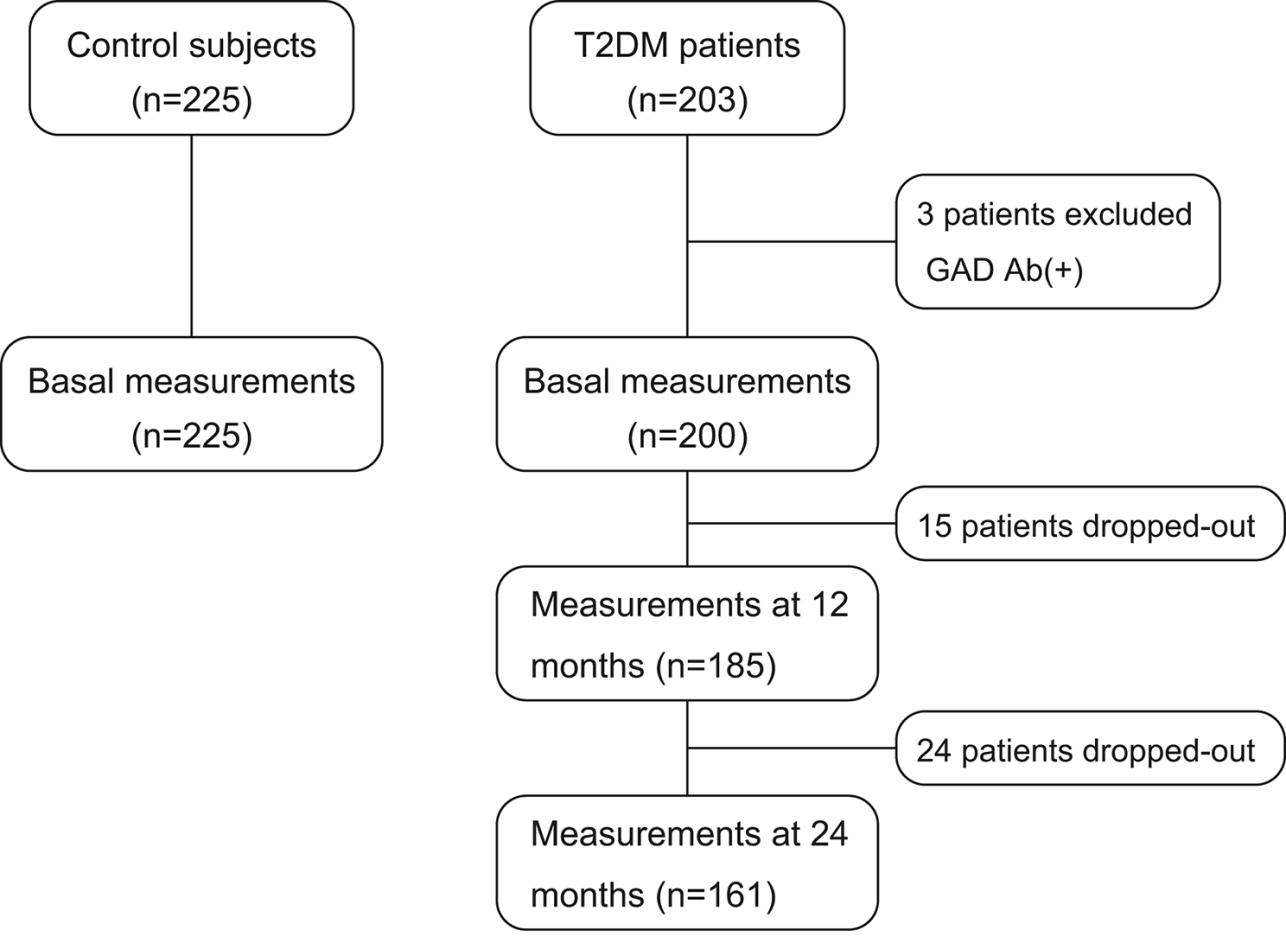
Patients flow chart

Included in this study were 203 patients with type 2 diabetes (127 males and 76 females) and 225 non-diabetic control subjects (115 males and 110 females) (Fig. 1). We excluded three patients with diabetes who showed GAD antibody positivity. Patients with type 2 diabetes were significantly older than control subjects and had significantly higher weight, BMI, systolic blood pressure, HbA1c, fasting plasma glucose, serum insulin, CPR, and triglyceride levels and significantly lower height, diastolic blood pressure, serum total cholesterol, HDL cholesterol, and LDL-cholesterol levels (Table 1). A significantly higher percentage of patients with type 2 diabetes were on medication for hypertension and dyslipidemia (Table 1). Most patient with type 2 diabetes (88%) were treated with anti-diabetic drugs including insulin (23%) (Table 1).

**Table 1:** Baseline characteristics and medications

	Control		T2DM	
**Characteristics**				
Male	115		124	
Female	110		76	
Total	225		200	
Age	51.0 ± 11.2		68.3 ± 10.3**	
Height(cm)	164.0 ± 8.6		161.3 ± 9.0**	
Weight (kg)	62.4 ± 12.6		68.8 ± 45.9*	
BMI (kg/m^2^)	23.1 ± 3.5		26.3 ± 17.1**	
Systolic BP (mmHg)	125.0 ± 16.3		133.9 ± 19.3**	
Diastolic BP (mmHg)	75.4 ± 11.4		73 ± 11.6*	
HbA1c(%)	5.4 ± 0.3		7.2 ± 0.8**	
FPG (mg/dl)	91.6 ± 8.5		147.9 ± 43.4*	
IRI (µU/ml)	6.8 ± 3.8		17.6 ± 32.3**	
CPR (ng/ml)	1.5 ± 0.6		2.5 ± 1.6**	
T-CHO (mg/dl)	217.7 ± 41.3		190.7 ± 36.2**	
TG (mg/dl)	106.7 ± 86.8		138.3 ± 88.2**	
HDL (mg/dl)	68.9 ± 15.6		54 ± 14.1**	
LDL (mg/dl)	124.5 ± 32.9		107.2 ± 26.8*	
**Medications**				
	number	(%)	number	(%)
For hypertension				
any	28	12.4	104	52.0**
ARB	16	7.1	79	39.5**
ACEI	0	0	5	1.5*
CaB	24	10.7	65	32.5**
α-blocker	1	0.4	5	2.5
β-blocker	4	1.8	4	2.0
diuretics	1	0.4	5	2.5
For dyslipidemia				
any	14	6.2	79	39.5*
statins	11	4.9	73	36.5*
ezetimib	1	0.4	5	2.5
fibrate	1	0.4	5	2.5
vitamin E	2	0.9	0	0
For diabetes				
any	-	-	176	88
DPPIVI	-	-	116	56.9
biguanide	-	-	91	45.5
SU	-	-	69	34.5
αGI	-	-	30	15
SGLT2	-	-	16	8
TZD	-	-	5	2.5
glinide	-	-	5	2.5
insulin	-	-	46	23
GLP1	-	-	7	3.5

**: *P* < 0.01, *: *P* < 0.05

ARB: angiotensin II receptor blocker, ACEI: angiotensin converting enzyme inhibitors

CaB: calcium channel blocker, DPIVI: dipeptidyl peptidase-4 inhibitor

SU: sulfonyl urea, αGI: α-glycosidase inhibitor

TZD: thiazolidinedione, GLP1: glucagon-like peptide 1 analog

### Thyroid function

Serum TSH levels of patients with type 2 diabetes were not significantly different from those of non-diabetic controls (Table 2). Serum fT3 were lower and fT4 levels were higher, however, in patients with type 2 diabetes compared with non-diabetic controls (Table 2). Accordingly, the fT3/fT4 ratio was significantly lower in patients with type 2 diabetes (Table 2). The number of patients with thyroid dysfunction (defined as thyrotropin or thyroid hormone levels out of reference range; normal reference range TSH:0.61–4.23 µIU/ml [35], fT3:1.72–3.44 pg/ml, fT4: 0.71–1.69 ng/dl, provided by manufacturer) among patients with type 2 diabetes were not significantly different from those among the control subjects (Table 2). The positive rates of thyroid autoantibodies were not significantly different between patients with type 2 diabetes and controls (Table 2).

**Table 2:** Thyroid function and autoantibodies

	Control		T2DM	
**Mean value**				
TSH (µIU/ml)	2.21 ± 0.4		2.14 ± 0.11	
fT3 (pg/ml)	2.49 ± 0.02		2.42 ± 0.30*	
fT4 (ng/dl)	1.14 ± 0.01		1.25 ± 0.21**	
fT3/fT4 ratio	2.23 ± 0.03		2.00 ± 0.03**	
**Thyroid dysfunction**				
	number	(%)	number	(%)
any	32	14.2	28	14
overt hyper	2	0.9	4	2.0
subclinical hyper	13	5.8	9	4.5
overt hypo	3	1.3	2	1.0
subclinical hypo	13	5.8	13	6.5
**Thyroid autoantibodies**				
	number	(%)	number	(%)
any	40	17.8	34	17
TgAb	30	13.3	32	16.0
TPOAb	18	8.0	14	7.0
TRAb	5	2.2	2	1.0

**Fig. 2:**
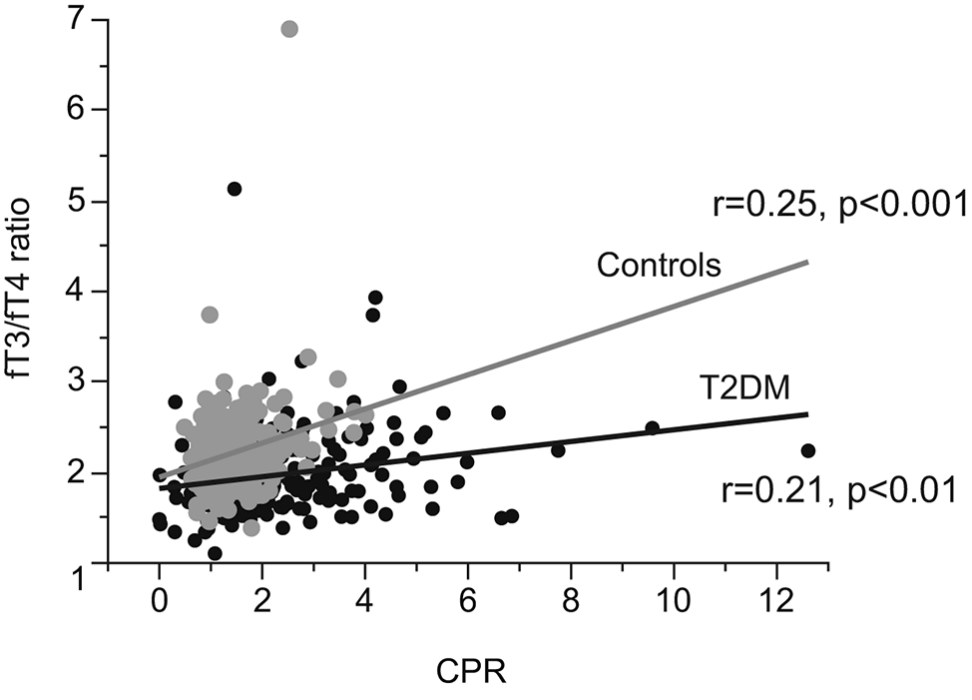
The relationship between fT3/fT4 ratio and CPR. Gray dot and gray line: controls. Black dot and black line: patients with type 2 diabetes (T2DM). CPR: serum c-peptide levels

To reveal the variables responsible for the differences of fT3 and fT4 levels between patients with type 2 diabetes and controls, multiple regression analysis with backward stepwise selection was conducted using valuables as follows: age, sex, BMI, TSH, fT3 (for T4 analysis), fT4 (for fT3 analysis), TgAb, TPOAb, TRAb, HbA1c, glucose, CPR, and osteocalcin. Serum fT4 levels were positively correlated with fT3 and HbA1c, and they were negatively correlated with TSH, CPR and osteocalcin (Table 3). Serum fT3 levels were positively correlated with fT4, TRAb, and CPR, and they were negatively correlated with age and male sex (Table 3). Serum fT3/fT4 ratio were positively correlated with TSH, CPR, and osteocalcin, and they were negatively correlated with HbA1c and age (Table 3). The fT3/fT4 ratio was significantly lower and CPR levels were higher in patients with type 2 diabetes, so the positive relationship between CPR levels and fT3/fT4 ratio seemed apparently reversed. This can be explained by the difference of correlation slopes between patients with type 2 diabetes and controls (Fig. 2).

**Table 3:** Correlation with fT4, fT3, fT3/fT4 ratio

	Standard regression coefficient	*P* value
**Correlation with fT4**		
TSH	-0.51	< 0.0001
fT3	0.53	< 0.0001
HbA1c	0.16	< 0.0001
CPR	-0.15	0.002
osteocalcin	-0.09	0.006
**Correlation with fT3**		
fT4	0.55	< 0.0001
TRAb	0.51	< 0.0001
CPR	0.34	< 0.0001
age	-0.2	< 0.0001
sex (male)	-0.05	0.0001
**Correlation with fT3/fT4 ratio**		
TSH	2.53	< 0.0001
CPR	0.41	< 0.0001
HbA1c	-0.23	< 0.0001
osteocalcin	0.20	0.0022
age	-0.13	0.018

Twelve months after the basal measurements, 15 diabetes patients dropped out, the remaining 185 patients with type 2 diabetes were still followed. Levothyroxine had been initiated in one patient who had TSH level of 11.03 µIU/ml at the first measurement. Methimazole had been prescribed to one patient who had been diagnosed with Graves’ syndrome after the first measurement. After omitting these two cases, mean TSH levels and fT3 levels at 12 months later were significantly elevated (2.08 ± 0.11 to 2.42 ± 0.14 µIU/ml, *P* < 0.01, 2.42 ± 0.02 to 2.53 ± 0.02 pg/ml, *P* < 0.01, respectively), whereas mean fT4 levels were significantly decreased (1.26 ± 0.02 to 1.18 ± 0.02 ng/ml, *P* < 0.01).

Twenty-four months after the basal measurements, 161 patients with type 2 diabetes were still followed. Mean fT3 levels at 24 months later were significantly decreased (2.53 ± 0.02 to 2.47 ± 0.02µIU/ml, *P* < 0.05), whereas mean fT4 levels were significantly elevated (1.17 ± 0.02 to 1.23 ± 0.02 ng/ml, *P* < 0.01) when compared to those at 12 months. Mean TSH levels were not changed during this period.

### Thyroid function and glycemic control

Basal HbA1c levels were not correlated with basal TSH, fT3, fT4 or fT3/fT4 ratio in patients with type 2 diabetes (data not shown). There was no significant correlation between basal TSH, fT3, fT4 or fT3/fT4 ratio with the amounts of changes of HbA1c levels at 12 or 24 months after the basal measurements (data not shown).

### Thyroid function and nephropathy

At baseline measurements, TSH levels were negatively correlated with eGFR levels (r=-0.29, *P* < 0.001). By multiple regression analysis with backward stepwise selection using valuables (age, sex, BMI, systolic blood pressure, diastolic blood pressure, TSH, fT3, fT4, TPOAb, TgAb, TRAb, glucose, HbA1c, CPR, osteocalcin, ferritin, and urine albumin/g‧cre), TSH levels were still negatively correlated with eGFR levels, respectively (Table 4). Twenty-four months later, mean eGFR levels were declined from 69.5 ± 1.58 to 63.3 ± 1.47 ml/min/1.73m^2^. TSH and fT3 levels were negatively (r=-0.26, *P* < 0.001) and positively (r = 0.26, *P* < 0.001) correlated with eGFR levels, respectively. By multiple regression analysis, however, these correlations were no longer observed. There was no significant correlation between basal thyroid tests with the amounts of changes of eGFR levels at 24 months after the basal measurements (data not shown).

**Table 4:** Correlation with eGFR at baseline

	Standard regression coefficient	*P* value
age	-24.60	< 0.0001
TSH	-17.00	0.0016
osteocalcin	-15.50	< 0.0001

Mean urine albumin/g‧cre levels were not changed for 24 months periods (188.2 ± 68.6 to 188.8 ± 46.8 mg/g‧cre). No relationships between thyroid function tests with urine albumin/g‧cre levels were observed at either baseline, 12, or 24 months later (data not shown).

## Discussion

In this study of thyroid function in patients with type 2 diabetes and controls, there were no differences in the rate of patients with thyroid dysfunction. The prevalence of thyroid disease has been widely reported to be high in patients with type 2 diabetes[2, 7, 8, 11, 13, 16, 19]. Although some studies did not strictly exclude patients with type 1 diabetes [2, 7, 8, 16, 19], others lacked control subjects [2, 19]. After excluding these reports, just one report clearly demonstrates that the higher prevalence of thyroid dysfunction among patients with type 2 diabetes than in controls, 7.3% vs. 2.9% [11]. Our findings are more consistent with those of several other studies [15, 21–23] that showed no significant differences in the prevalence of thyroid dysfunction between patients with type 2 diabetes and controls.

Recently, Peters et al. examined the prevalence of thyroid dysfunction in Australian patients with diabetes including type 1, type 2 and latent autoimmune diabetes, and provided detailed background information [36]. The prevalence of subclinical hypothyroidism, overt hypothyroidism, subclinical hyperthyroidism, and overt hyperthyroidism were 4.9%, 1.2%, 0.1%, and 0.2%, respectively, in patients with type 2 diabetes after excluding patients with known thyroid disease [36]. Compared with their data, our data showed higher incidence of subclinical (4.4%) and overt hyperthyroidism (2.4%). These differences may be attributable to the differences in subject’s characteristics, including race, age, and iodine intake. Regarding iodine intake, according to a WHO report in 2004, Australia is mildly iodine deficient region [37], while Japanese people intake a comparatively very high amount of iodine through consumption of seaweeds. Other possible factors might be the difference of thyroid function test kits and the adequateness of their reference range. Thyroid tests are not currently standardized, and the reference ranges of each kit are usually based on the 99% interval values of a relatively small number of healthy controls, making it difficult to define “thyroid dysfunction”, especially when it is subclinical.

We found that serum fT3/fT4 levels were significantly lower in patients with type 2 diabetes when compared to those in controls. FT3/fT4 ratio has been indicated to be influenced by insulin sensitivity [38, 39]. Ferrannini et al. found that fT3/fT4 ratio negatively correlated with insulin sensitivities determined by insulin-clamp in 940 non-diabetic patients with normal thyroid function [38], while Park et al. found that fT3/fT4 ratio corelated with metabolic syndrome parameters and insulin resistance in 132,346 subjects who underwent medical health check-up programs [39]. Our data that fT3/fT4 ratio is lower in patients with type 2 diabetes was in accordance with these reports. The mechanism by which fT3/fT4 ratio is influenced by insulin sensitivity is not completely understood. Loss of insulin action was indicated to be suppressed T3 production from T4 in rats [40]. Although mean serum CPR levels were even more significantly higher in patients with type 2 diabetes in our study, their action might have been decreased due to insulin resistance. The correlation between fT3/fT4 ratio and serum CPR levels being less pronounced in patients with type 2 diabetes may support this idea. Very weak association between fT3/fT4 ratio and osteocalcin levels were also observed. Osteocalcin is used as a marker for bone turnover in the clinical setting. It has been also suggested to be involved in the regulation of glucose homeostasis [41] and relationship between serum osteocalcin levels and insulin secretion was reported [42]. The observed relationship between fT3/fT4 and osteocalcin levels may be indirectly related to insulin levels.

Association between thyroid dysfunction and diabetic complications including nephropathy[29–31] and retinopathy, has been shown [32, 33, 43], although some studies found no relationship between thyroid dysfunction and retinopathy [29, 44]. In this study, we found no relationship between thyroid function with urine albumin/g‧cre levels, whose levels increase with the development of diabetic nephropathy. Previous reports showed the negative relationship between TSH and eGFR levels among individuals with normal kidney function or with chronic renal disease (CKD) [45, 46]. We found negative relationship between TSH levels with eGFR at baseline measurements, which was in accordance with these reports. However, TSH levels did not seem to predict future decline of eGFR levels. Other factors related to the eGFR levels were age and osteocalcin levels, both of which were negatively correlated with eGFR levels. Osteocalcin is a marker for bone turnover, which is mainly excreted by kidney [47]. The observed relationship between eGFR and osteocalcin levels may reflect decreased excretion of osteocalcin or increased bone turnover associated with CKD (chronic kidney disease)-MBD (mineral and bone disorder).

Due to the study protocol, this study has several limitations. First, the basal characteristics of patients with diabetes and control subjects were quite different; subjects receiving medical checkups are generally healthy and relatively young. The prevalence of thyroid dysfunction increases with age, especially for hypothyroidism [48], so it might have caused bias for lower prevalence rate of hypothyroidism among control subjects who were significantly younger than patients with diabetes. Second, we excluded patients with previously-known thyroid disease at the time of enrollment. Our institute is specialized for both thyroid disease and diabetes, so it is not unusual that patients referred to our hospital due to thyroid disease also have diabetes. We considered that this might cause bias for higher prevalence of thyroid disease in patients with diabetes if we include the patients with diabetes who had previously-known thyroid disease. Considering that higher percentage of patients with diabetes might have undergone screening thyroid tests in our hospital compared with control subjects, excluding patients with previously-known thyroid disease might have caused bias for lower prevalence of thyroid disease in patients with diabetes. Third, the number of patients with diabetes with thyroid dysfunction were small due to the relatively small cohort. No relationship was found between baseline thyroid function and future diabetes control or renal function, which might be attributable to inadequate power due to the small number of thyroid dysfunctions. We did not restrict addition of diabetes medication, which also might have affected the results. The duration of diabetes was not considered in this study, due to the lack of precise data of diabetes history. There is a possibility that prevalence of thyroid dysfunction may be different among groups with different diabetes history.

## Conclusion

There were no differences in prevalence of thyroid dysfunction and thyroid autoantibodies between patients with type 2 diabetes and controls, although the ratio of fT3/fT4 was decreased in patients with type 2 diabetes. Basal thyroid function did not predict future diabetes control or renal function within a 24-month period.

## Data Availability

The datasets used and/or analyzed during the current study are available from the corresponding author on reasonable request.
